# The Italian public health response during the pandemic emergency: from qualitative data to the “performance index” of care provided by Spoleto Hospital

**DOI:** 10.3389/fpubh.2025.1337375

**Published:** 2025-06-16

**Authors:** Silvia Caroselli, Martina Roselli, Aldo Germani, Marco Fabiani, Caterina Micolonghi, Vincenzo Visco, Salvatore Raffa, Rita Mancini, Simona Petrucci, Alexander Cardone, Orietta Rossi, Giorgio Banchieri, Christian Napoli, Maria Piane

**Affiliations:** ^1^Department of Experimental Medicine, Sapienza University of Rome, Rome, Italy; ^2^Juno Genetics, Reproductive Genetics, Rome, Italy; ^3^San Matteo degli Infermi Hospital of Spoleto, Spoleto, Italy; ^4^Department of Clinical and Molecular Medicine, Sapienza University of Rome, Rome, Italy; ^5^ALTAMEDICA, Human Genetics, Rome, Italy; ^6^Sant’Andrea University Hospital, Rome, Italy; ^7^Associazione Italiana per la Qualità della Assistenza Sanitaria e Sociale (ASIQUAS), Largo Konrad, Adenauer 1/B, Rome, Italy; ^8^DiSSE, Department of Economic and Social Sciences, Sapienza University of Rome and LUISS Business School, Rome, Italy; ^9^Department of Medical Surgical Sciences and Translational Medicine, Sapienza University of Rome, Rome, Italy

**Keywords:** COVID-19, SWOT analysis, healthcare management, Streetlight PRIority Swot system (SPRIS), priority score, performance index

## Abstract

**Background:**

The public health emergency was one of the most severe consequences of the COVID-19 pandemic outbreak, which occurred in successive waves since March 2020. In this scenario, the Hospital of Spoleto “San Matteo degli Infermi” (located in the Umbria region, Italy) became a COVID-19 referral center and therefore had to make organizational changes. This study aims to evaluate the quality of care provided during the pandemic and to explore what the hospital management should focus on.

**Methods:**

An online survey related to ten topics across the five pandemic waves that took place in Italy from March 2020 to February 2022, was administered to the hospital unit referents. The qualitative responses collected were analyzed quantitatively using a recognized tool, called “Streetlight PRIority Swot” (SPRIS) system and based on a new and multilevel “strengths, weaknesses, opportunities, threats” (SWOT) matrix.

**Results:**

It was highlighted that the demand for continuity of care for patients and an increase in personal protective equipment were the issues that should have been the focus of the intervention after the first wave. Taking this into account, an improvement in performance was observed in the subsequent waves. Therefore, the results described a more than good quality of care provided among the hospital units, although with the need to improve the orthopedic services, emerged as the most critical area.

**Limitations, reasons for caution:**

Due to practical limitations, the study population was limited to the hospital unit referents. Future broader surveys may enrich the information from the hospital experience. The SPRIS system uses a general-to-specific approach which can lead to a complex outcome assessment. However, careful and continuous application supports the analytical validity and utility of this method.

**Conclusion:**

The analysis based on the SPRIS system showed the effective response of Spoleto Hospital after the first sudden wave for the following four pandemic waves, driven by the implementation of safety measures. The perspective adopted and the scenario tested can be seen as a starting point for an educational tool to monitor and evaluate health management strategies during emergency periods.

## 1 Introduction

The SARS-CoV-2-related disease has negatively impacted our society in all its spheres, without precedent in contemporary history (1, 2). It was first reported at the end of December 2019, during an outbreak that emerged in China and rapidly spread around the world (3). On 30th January 2020, the World Health Organization (WHO) declared the COVID-19 outbreak as a public emergency of international concern (4) and as a pandemic on 11th March 2020, alerting all countries to immediate notice and action (5). In Italy, the COVID-19 pandemic occurred from late February to early March 2020 (6), resulting in subsequent up and down periods (waves) in the number of cases (i.e., time-points of spread and containment of infections, respectively) (7, 8). To date, the global impact of the pandemic has been profound, with over 770 million confirmed cases and more than 7 million deaths reported worldwide, including over 26.9 million cases and approximately 198,638 deaths in Italy (9). At the beginning of the pandemic, five waves have been distinguished (10, 11): (i) March – June 2020; (ii) October 2020 – January 2021; (iii) February – June 2021; (iv) July – October 2021; (v) November 2021 – March 2022. During this period, the pandemic crisis particularly strained the Italian National Health System (I-NHS) at several levels and proved one of the most demanding challenges it ever faced (12). The spread of the pandemic caused prolonged periods of stress and high emotional load on human resources. All of this also affected the health status and the psycho-physical well-being of healthcare workers through extended working hours and continuous exposure to the virus (13, 14). In terms of health-care resources, the growing demand for COVID-19 treatment exceeded “normal” emergency surge capacity, defined as the ability of a hospital to expand care for a sudden dynamic influx of patients. This had to be managed in a short period of time (15), while maintaining the health-care support for non-SARS-CoV-2-related diseases (16). To address this emergency, the I-NHS redesigned its network, resulting in the conversion of hospitals and local health centers. Moreover, structural (e.g., increasing treatment space) and organizational (e.g., cancelation of elective surgeries) changes (17) were made to ensure the well-being of both patients and staff. Several studies assessed the impact of the COVID-19 pandemic on specific areas, such as neuromuscular (18) and chronic liver (19) care units and surgical services (20, 21). Currently, there is a lack of evidence on how problems were manifested during the COVID-19 pandemic, or what was done to address these challenges at the hospital-care level, as a complex network involving multiple medical facilities. To support this need, the experience of those working in the hospital is a very valuable contribution to the policy-making process. Therefore, gathering this evidence through interviews or survey results is one of the best ways to evaluate the quality of care and the policy itself (22). Quality of care is the degree to which health services for individuals and populations increase the likelihood of desired health outcomes. To achieve the highest possible quality of care, a framework for improving the ways care is delivered to patients is essential. Therefore, specific tools and methods have been proposed for interpreting the survey results and drawing meaningful conclusions (23). Among these, the SPRIS (Streetlight PRIority Swot) system, based on a multilevel “strengths, weaknesses, opportunities, threats” (SWOT) matrix, has recently been developed (24, 25).

Our objectives were to evaluate the quality of health services provided over the first pandemic waves by the hospital of Spoleto “San Matteo degli Infermi” and to highlight the improvement actions needed to respond to the challenges posed by the COVID-19 pandemic. Indeed, this hospital has been converted into a center dedicated to COVID-19 by regional ordinance (26), playing a strategic role in the regional health network and representing an interesting case study.

## 2 Methods

### 2.1 Study setting, design and participants

This observational study was conducted at Spoleto Hospital, which is located in Umbria, a region in central Italy. It is composed of three Organizational Articulations (OA): Inpatient Units, Diagnosis and Care Services and Hospital Polyclinics, respectively divided into 7, 14 and 7 Operational Units (OUs) (Table 1).

**Table 1:** Medical facilities enrolled.

Organizational Articulations (OAs)					
UOs (OA1)	Inpatient units (OA1)	UOs (OA2)	Diagnosis and care services (OA2)	UOs (OA3)	Hospital polyclinics (OA3)
A	General Medicine	A	Pathological Anatomy	A	Audiology, Phoniatrics and Ear-nose-laryngology
B	Onco-haematology	B	Anesthesiology	B	General Surgery
C	General Surgery	C	Angiology	C	Orthopaedics
D	Obstetrics and Gynecology	D	Cardiology	D	Paediatrics
E	Ophthalmology	E	Dietetics	E	Hospital Polyclinics
F	Orthopaedic- Traumatology	F	Gastrointestinal Endoscopy	F	Accident and Emergency
G	Reanimation	G	Haepatology	G	Pain Therapy
		H	Analysis Laboratory		
		I	Nephrology and Dialysis		
		J	Neurophysiopathology		
		K	Radiology		
		L	Radiotherapy		
		M	Cardiovascular Rehabilitation		
		N	Transfusional and immunological Medicine		

The Organizational Articulations (OAs) network is subdivided into several Operational Units (OUs). The 28 respondents to the survey are referents of each of the OUs listed in the table with correspondences of their reference alphanumeric identifiers.

The period analyzed runs from February 2020 to March 2022, divided into five pandemic waves, identified in Italy by the *Istituto Superiore di Sanità* (7) and the Italian Department of Civil Protection (8) on the basis of the incidence and prevalence of cases recorded at the national level (10, 11): (i) 27th February 2020 – 28th June 2020; (ii) 1st October 2020 – 2nd February 2021; (iii) 26th February 2021 – 5th July 2021; (iv) 14th July 2021 – 11th October 2021; (v) 23rd October 2021 – 31st March 2022.

From October to November 2022, 28 referents for each OU of Spoleto Hospital were invited to participate in a cross-sectional survey, to obtain a complete snapshot of the experience. As supervisors or coordinators, these referents were also information-richer and more available respondents (27, 28).

#### 2.1.1 Ethical considerations

The study was conducted according to the guidelines of the Declaration of Helsinki and approved by the Institutional Review Board (or Ethics Committee) of Sapienza University of Rome, Italy (RIF. CE 5773_2020, Prot. #52SA_2020, and Prot. #171SA_2020). We have taken all necessary measures to ensure the anonymity and confidentiality of the participating physicians’ data. No personally identifiable information was included in our research and will not be disclosed to third parties. Data has been handled in accordance with art. 13 reg. EU 679/2016 (GDPR). Specifically, on the first page of the questionnaire, the respondents were informed about the purpose of the study and the data policy, after which they gave their informed consent for access.

### 2.2 Survey questionnaire — methodological approach and data collection

The questionnaire was specifically developed based on guidance from government documents, published literature, and best practices (23, 29–31). It provided multiple-choice answers for each query, with a worded rating scale for feedback options: yes, enough, not enough, not at all and “not applicable” if the item was not relevant. Respondents were asked to indicate the qualitative category that comes closest to their position, coding the responses in a more homogeneous manner. Later, these qualitative results were converted into quantitative data using a Likert scale from 4 to 1 (32); excluding responses marked as “not applicable.”

**Figure 1:**
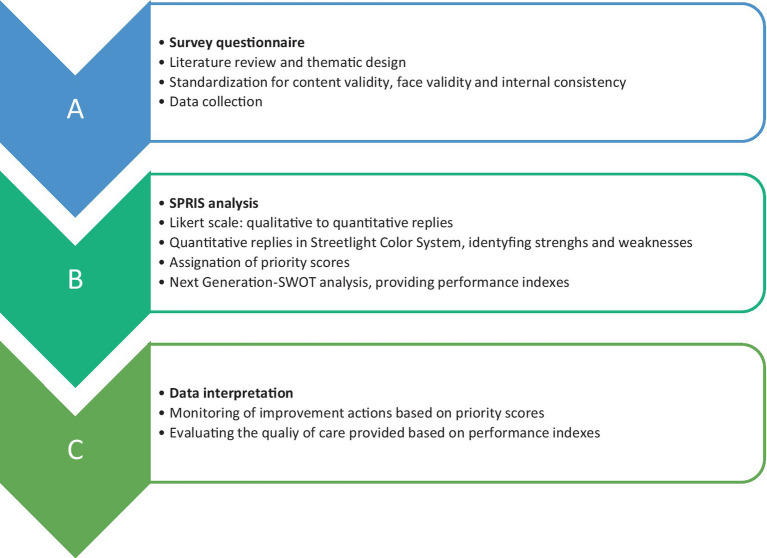
Workflow chart for the application of SPRIS analysis to this Italian public health setting. For methodological approach used for survey design and SPRIS system development, referring to (24, 25).

To ensure the scientific appropriateness of the questionnaire prior to its administration, an effective yet concise validation process was implemented (Figure 1). First, the questionnaire was reviewed by domain experts (an epidemiologist and two healthcare executives) to assess the relevance and completeness of the items. Their feedback confirmed that the content covered key areas related to hospital response during the COVID-19 pandemic, thus establishing expert-based content validity. Second, informal face validity was assessed by piloting the questionnaire with a nurse and a laboratory technician not involved in the study. Their suggestions helped to improve the clarity and readability of some items. Third, internal consistency was evaluated using Cronbach’s alpha calculated on the full set of items using IBM SPSS Statistics for Windows (version 19). It reached a value of 0.917, indicating excellent internal consistency reliability. The standardized item alpha was 0.913, further confirming that the items provide highly consistent responses across subjects.

The final version of the questionnaire consisted of 81 items divided into ten sections according to thematic areas. For the sake of completeness, this version translated from Italian into English, is presented in Table 2. It was administered to the 28 referents of each of the OUs of the three Organizational Articulations of Spoleto Hospital using the “Microsoft Forms” platform (Microsoft Office 365, 2021).

**Table 2:** Structure and content of the survey administered.

Section number	Section title	Sub-sections	Items
1	Context Analysis	1	5
2	Patient Access to the hospital	2	10
3	Impact on taking charge of non-COVID-19 patients	2	2
4	Impact on taking charge of COVID-19 patients	2	10
5	Impact on patient management	2	10
6	Experience at COVID-19 referral centre	6	6
7	Procedures and recommendations for healthcare personnel/users	2	10
8	Education-Information-Training: healthcare professionals’ management	2	10
9	Analysis of factors internal to the organization	10	10
10	Analysis of factors external to the organization	8	8
			Total: 81

### 2.3 Data analysis

The collected quantitative dataset was analyzed using the SPRIS system (24, 25), as summarized in Figure 1. First, the Streetlight color system is a graphic model with a colored scale, namely green for values of 4 and 3, yellow for 2, red for 1 and gray for 0. This tier thus displays the results, providing an immediate snapshot of the experience of Spoleto hospital and allowing users to identify the critical issues. Later, the SPRIS system processes the quantitative data collected from the questionnaire to assign and calculate two parameters: the Priority Score and the Performance Index, both of which can be used in strategic planning for improvement and monitoring.

#### 2.3.1 Priority score

The Priority Score is a value assigned to each weakness or strength emerging from the survey, using a conversion scale (24) where there is a corresponding score for each range of quantitative results. In this way, it is possible to highlight whether improvement actions are needed and to indicate where and when to intervene (e.g., which section/OAs/UOs and in which order). In other words, the priority score numerically defines how important the query is in the strategic planning: (i) to prioritize improvement actions to be taken for weaknesses, (ii) to indicate the valuable impact for strengths, (iii) to build a decision matrix and timeline of interventions to improve the quality of the services provided.

#### 2.3.2 Performance index

The performance index is a measure of the quality of the activity/service provided by the OAs/UOs during the pandemic and is obtained by entering the Priority score in the *Next Generation SWOT Analysis* (23). As the survey is based on objective items only, the SWOT analysis presents two sets of elements (i.e., strengths and weaknesses). Finally, five ranges of the performance index were considered to evaluate the responses (23): (i) < 5 corresponds to “null,” (ii) > 5 and <30 to “low,” (iii) > 30 and <60 to “good,” (iv) > 60 and <80 to “high” and (v) > 80 to “very high.”

Overall, we performed the analysis at two levels of query aggregation: the deeper one for items and the shallower one for sections, thus obtaining two performance indexes for each respondent. In terms of respondents, the results were released cumulatively for all the Organizational Articulations, while for each Organizational Articulation and Operational Unit they were released separately.

## 3 Results

### 3.1 Survey findings and streetlight color system

**Figure 2:**
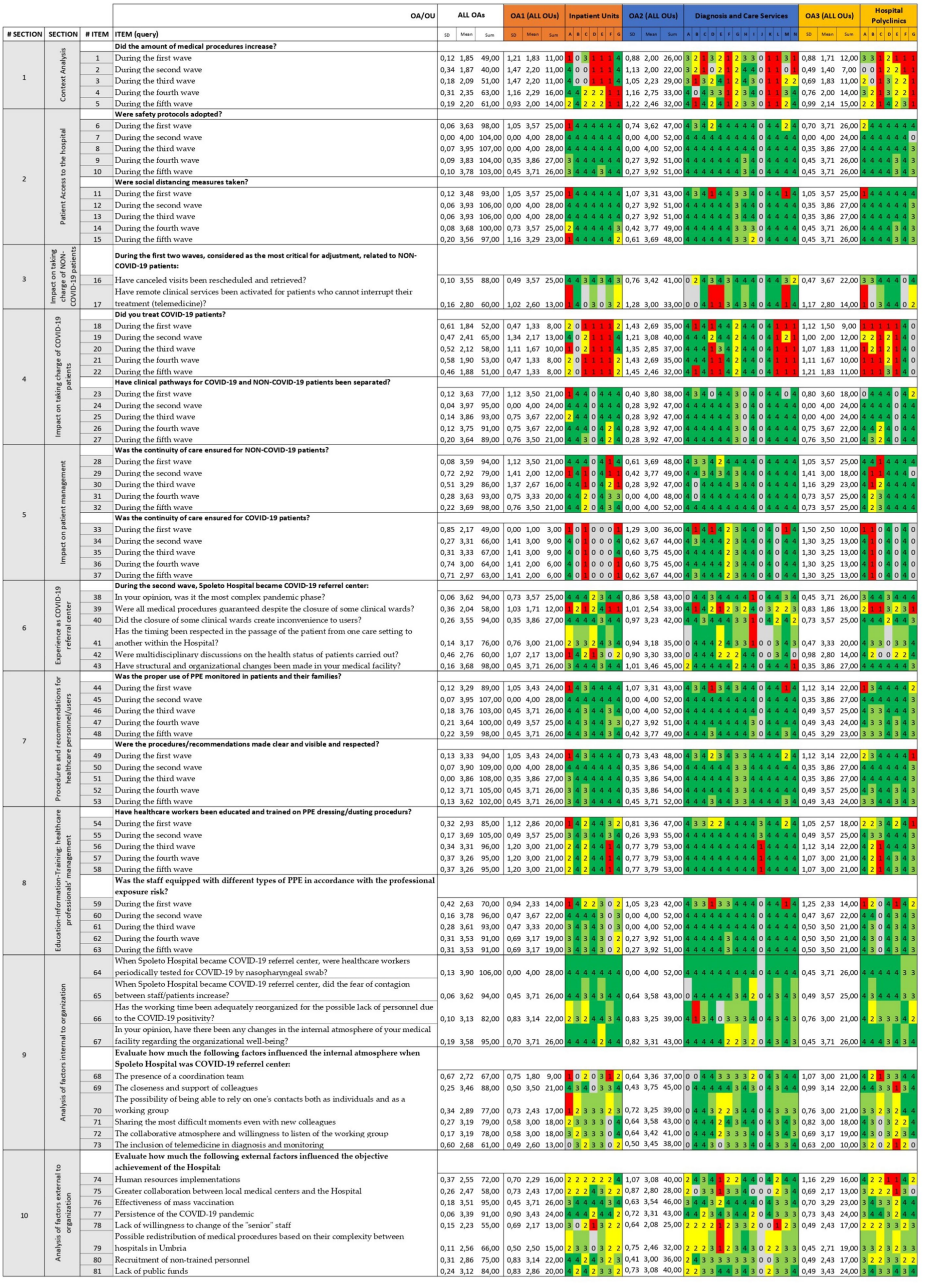
Summary of survey findings by the Streetlight color system (first tier). Quantitative results are identified by conversion scale: “yes” = 4 and “enough” = 3 are colored in green as strength, “not enough” = 2 is in yellow as faint weakness whilst “not at all” = 1 in red as strong weakness. The value “not applicable” = 0 is excluded and therefore colored in grey. Questionnaire items are listed here. Respondents (i.e., OA and OU individually) are referred to as their alphanumerical identifiers (see Table 1).

We collected 27 out of 28 completed questionnaires from the referents enrolled (*Neurophysiopathology*, OA2-J, was not available, “na”). For all queries, the qualitative results for each participant were converted into quantitative data and formatted using the *Streetlight colour system* (Figure 2).

### 3.2 Priority scores and the monitoring of improvement actions

**Figure 3:**
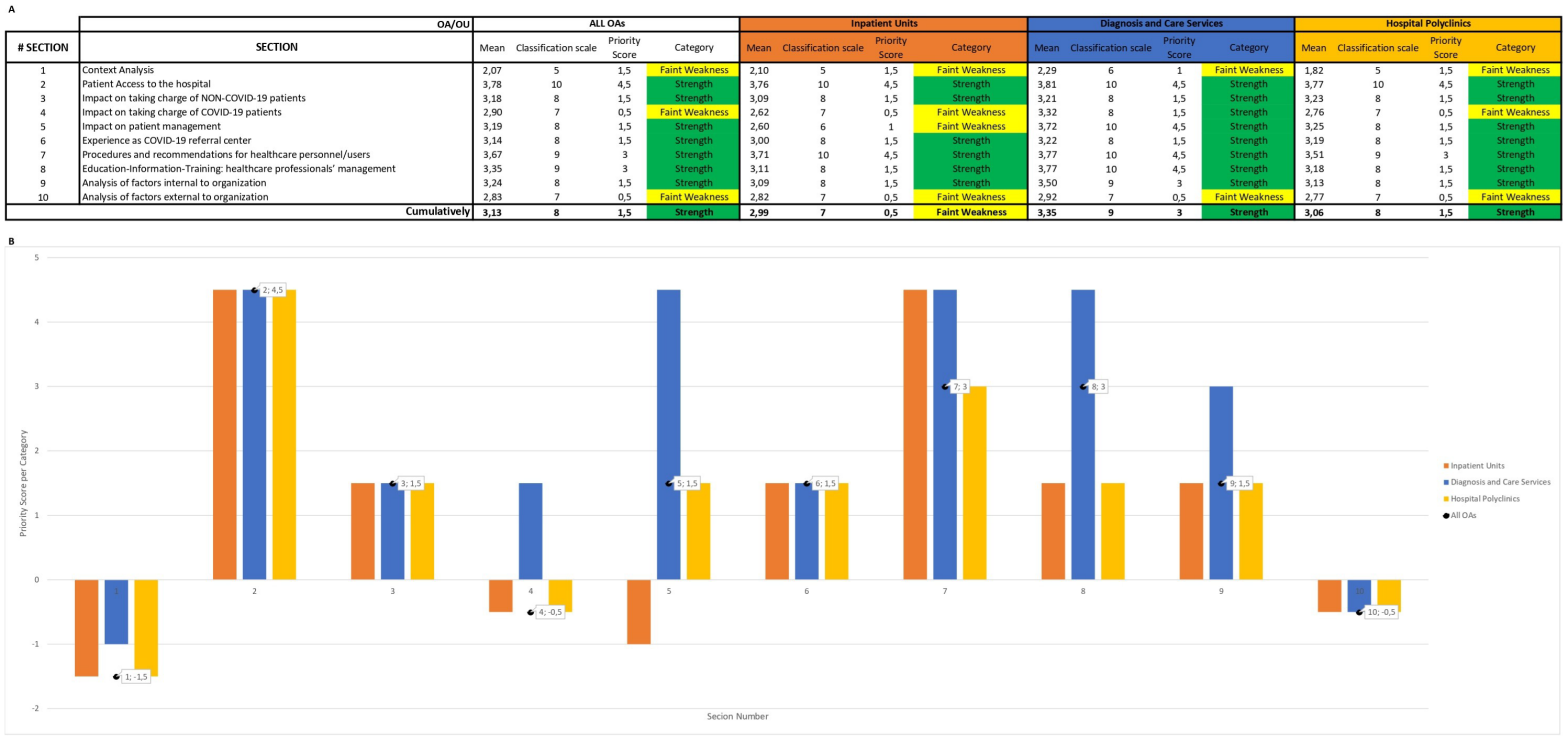
Summary of Priority score system (second tier). (A) Results related to sections for each OA, based on the conversion scale. (B) The bar chart shows the priority scores along *Y*-axis: negative values for weakness and positive ones for strengths.

**Figure 4:**
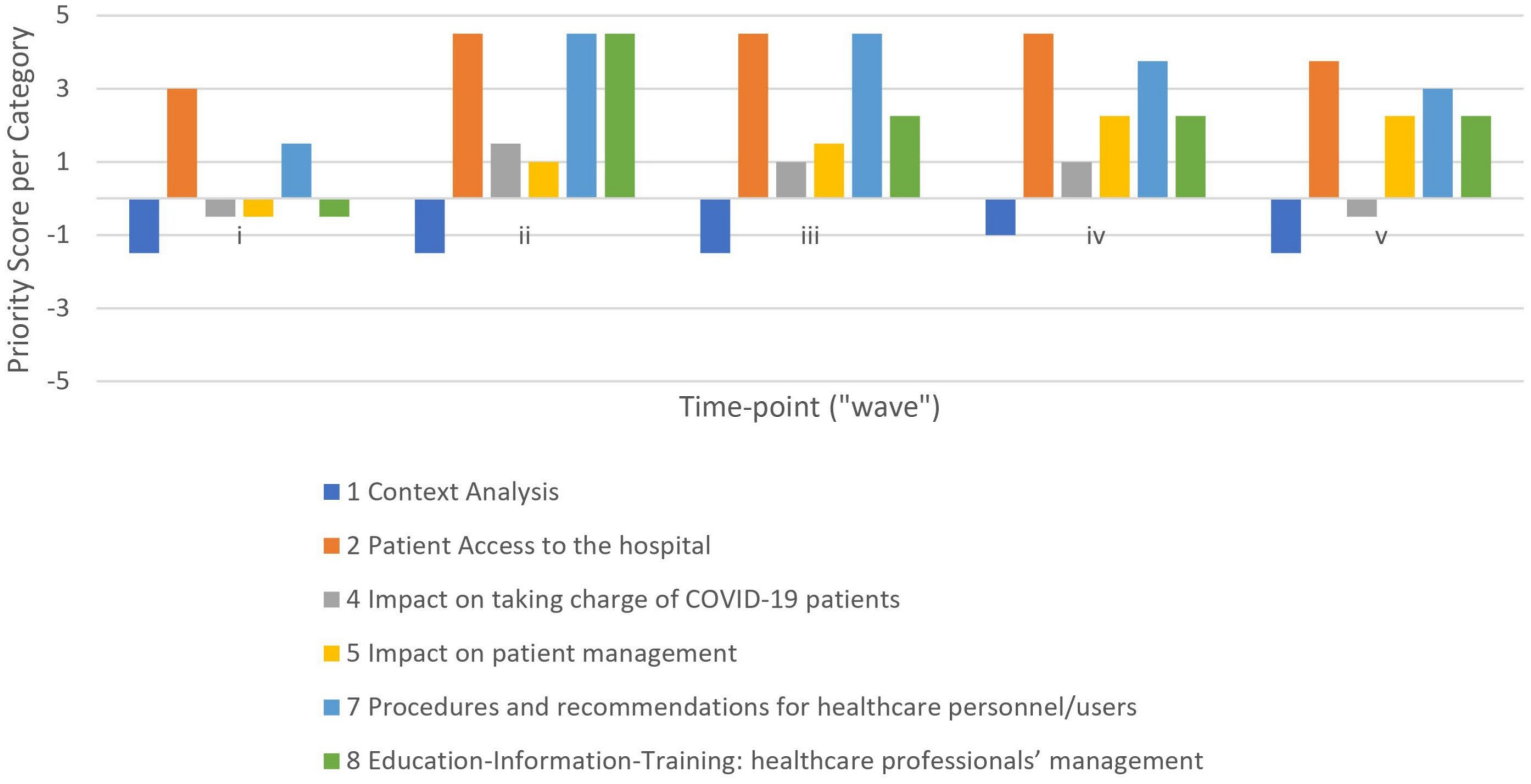
Category evolution during the first five waves. The bar chart shows the priority scores of sections included along the *Y*-axis: negative values for weakness and positive ones for strengths. Results related to respondents cumulatively.

By calculating the *priority scores*, we identified each section as a weakness or a strength. In particular, the survey sections 2, 3, 5, 6, 7, 8 and 9 resulted in strengths. In other words, “Patient Access to the hospital” was optimal by applying safety protocols and social distancing; “The taking charge of NON-COVID-19 patients” was sufficient by adapting their management (e.g., rescheduling appointments, telemedicine); “Patient management” was good by ensuring therapeutic continuity; “Experience as COVID-19 referral center” was sufficient; “Procedures and recommendations for healthcare personnel/users” and “Education-Information-Training: healthcare professionals’ management” were well applied; and finally “Factors internal to organization” had a positive influence in responding to the challenges of the pandemic (e.g., staff rotation, teamwork). The remaining sections (survey sections 1, 4, and 10) showed faint weaknesses. In particular, “Context Analysis” and “The taking charge of COVID-19 patients” showed that the volume of procedures was not increased excessively; and finally, “Factors external to organization” had a negative impact on the management of the public health emergency (e.g., mass vaccination, lack of stuff and funding). Considering the whole pandemic period, the results related to sections for each OA are described in Figure 3. In addition, Supplementary Tables 1–3 show the results related to items for both OAs and Ous individually. However, by applying the priority score system retrospectively, we were able to look at the results for each ‘wave’ (time-point) separately. In particular, we focused on six out of ten sections (i.e., survey sections 1, 2, 4, 5, 7 and 8), that could be examined during each pandemic wave, for a total of 11 sub-sections and 55 items. In this way, the priority scores obtained were evaluated in the context of strategic planning. As a result, it was proposed to the executive board to implement policies to ensure continuity of patient care and adoption of Personal Protective Equipment (PPE) after the first wave. By monitoring their impact, an improvement in the priority scores for section 5 and section 8 was noted, turning from faint weaknesses to strengths (Figure 4).

### 3.3 Performance indexes as evaluation of the conduct of medical facilities

Later, we defined the performance of the enrolled medical facilities by calculating *performance indexes (PI)* through the *Next-Generation SWOT Analysis* (Supplementary Figures 1–4). Cumulatively, Spoleto Hospital showed “very high” performance in both settings (i.e., query aggregation for items and sections), with PI equal to 87% and 86.8%, respectively, (Table 3).

**Table 3:** Summary of performances (third tier).

Respondent aggregation level	Respondent	Query aggregation level	Strengths	Weaknesses	Performance index (%)
(a) All the OAs	–	for items	174	26	87
		for sections	16	2.5	86.8
(b) for each OA	Inpatient Units	for items	166.5	42	79.9
		for sections	15	3.5	81.1
	Diagnosis and Care Services	for items	234	12	95.1
		for sections	25.5	1.5	92.7
	Hospital Polyclinics	for items	174	29	85.7
		for sections	15	2.5	85.7
(c) for each OU	Orthopaedic- Traumatology	for items	135	142	48.7
		for sections	9	13.5	40
	Reanimation	for items	226.5	54.5	80.6
		for sections	16.5	2.5	86.8
	Radiotherapy	for items	225	65.5	77.5
	Pathological Anatomy	for sections	25.5	11	69.9
	Angiology	for items	309	10.5	96.7
		for sections	37.5	0.5	98.7
	Orthopaedics	for items	148.5	134.5	52.5
	Hospital Polyclinics	for sections	7.5	10.5	41.7
	Accident and Emergency	for items	283.5	21.5	93
		for sections	27	3.5	88.5

Results for respondents by both the deeper and the shallower analysis.

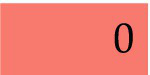
 
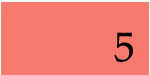
 
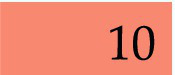
 
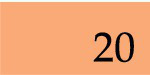
 
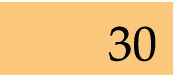
 
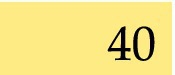
 
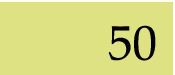
 
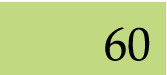
 
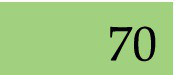
 
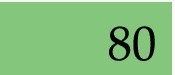
 
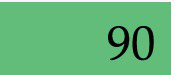
.

Looking at the OAs, *Inpatient Units*, *Diagnosis and Care Services* and *Hospital Polyclinics* also individually achieved “very high” performance range in both analyses, as well. The results for each OA are shown in Table 3. Considering the OUs, the results varied from “good” (>30–60%) to a “very high” performance range; highlighting the best and the worst nosocomial ward: *Angiology* and *Orthopaedic-Traumatology*, respectively. In addition, Table 3 shows the performance results for individual OUs.

**Figure 5:**
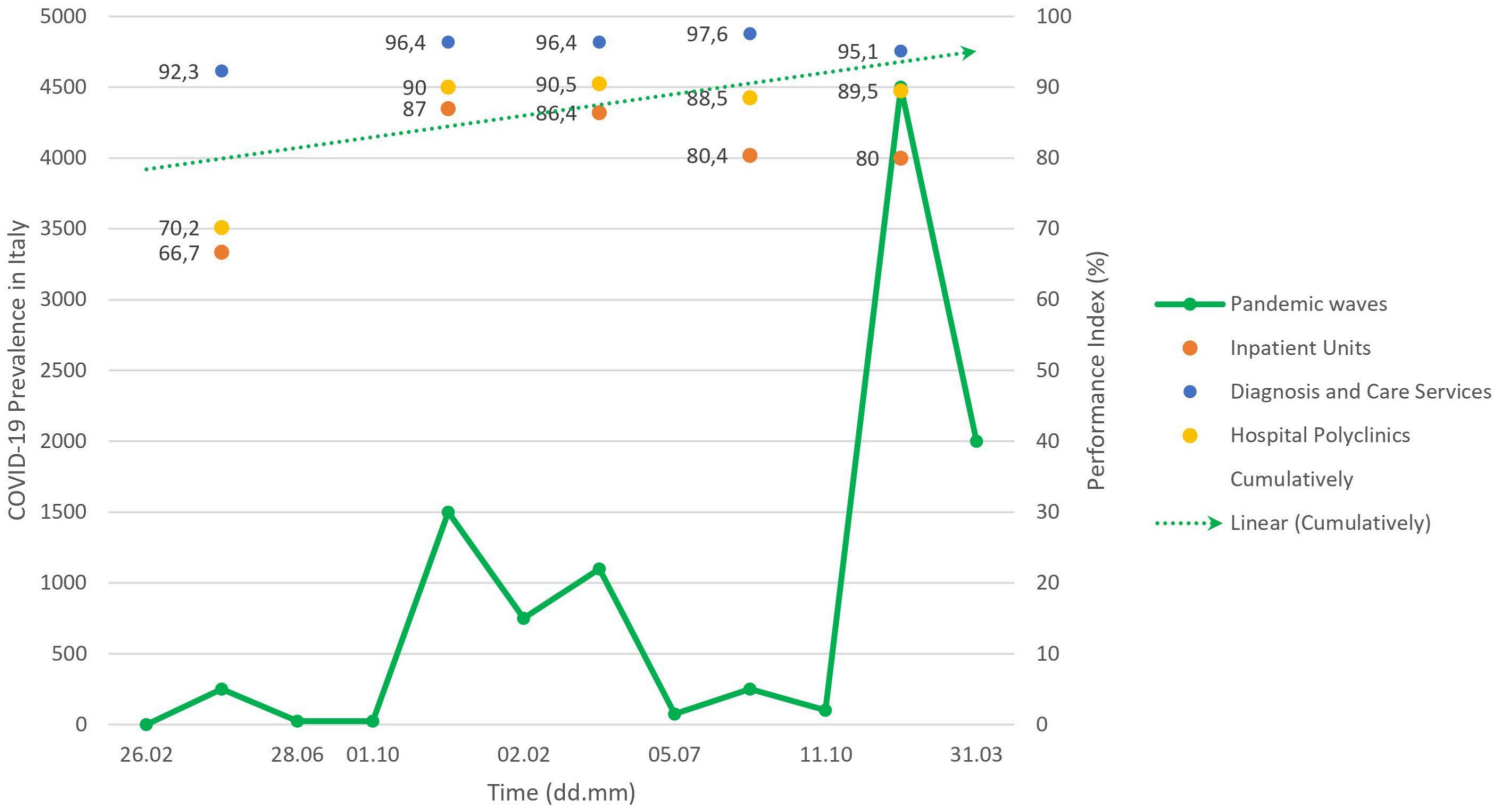
Performance trend during the first five waves.

Regarding the evolution over the waves, the improvement of the priority scores was reflected in an improving trend of the performance indexes for each OA during the subsequent pandemic waves (Figure 5, colored dots). Although the first sudden pandemic event resulted in an acceptable cumulative performance index of 73.2%; the situation was rapidly improved (Figure 5, dashed arrow), with “very high” performance always being achieved (i.e., overall performance indexes of 91.7%; 90.9%; 92.3% and 87.9% per time-point respectively). In addition, Supplementary Table 4 shows the results for individual OUs.

## 4 Discussion

In this study, we report an evaluation of the quality of care provided by Spoleto Hospital and of what the hospital management should have focused on during an emergency such as the COVID-19 pandemic.

Spoleto Hospital is an Italian public hospital belonging to the local health system USL-2 (i.e., *Azienda Unità Sanitaria Locale*) of the Umbria region with a catchment area of approximately 45,000 people. In this area, only one other hospital in Foligno, called “San Giovanni Battista,” was involved in pandemic management, with a mixed-care model and for the first wave. Conversely, Spoleto Hospital underwent a more comprehensive and prolonged reconfiguration, with most departments being dedicated to COVID-19 care over time. This marked structural and organizational change provided a consistent and well-defined context for analysis.

Since the COVID-19 pandemic is a multifaceted and rapidly evolving phenomenon, it was important to study its impact at the level of hospital-care (33, 34), as a complex network of several medical facilities, and during each of the five waves (35, 36).

First, it was possible to highlight how care pathways functioned during the overall COVID-19 pandemic, identifying strengths, weaknesses and needed interventions. The OAs achieved an optimal level of care (Table 3), indicating the appropriateness of the approaches taken. Considering the results for each OU, *Angiology* (ward C of OA2) performed best and *Orthopaedic-Traumatology* (ward F of OA1) performed worst (Table 3). This reflects the pathogenesis of COVID-19 disease and its epidemiology. Indeed, Coronavirus disease predisposes patients to arterial and venous thrombotic complications (37), and therefore the management of patients with pre-existing cardiovascular disease and infected patients who develop thrombosis, had to be dramatically faced and protected by the *Angiology Unit* (38, 39). On the contrary, orthopedic and trauma surgery are not disciplines directly involved in the clinical management of COVID-19 patients. Moreover, the rate of trauma and fragility fractures appeared to decrease during the pandemic era (40), showing significant temporal associations with daily population mobility and social distancing measures. Nevertheless, strategic planning of improvement actions in orthopedic services is needed, as confirmed by the literature (41–43).

A comparison of conditions over time was also made (Figure 2). Briefly, the first wave had a lower prevalence and duration than the others (10), although it was the wave that placed the greatest burden on the health-care system because it was caught off guard and did not have emergency management protocols and procedures in place (44). In light of this, the national lockdown was introduced as a containment strategy (30). After the loosening of containment measures, there were two tight and higher waves between autumn 2020 and spring 2021 (10). However, a lower case-fatality rate (CFR, i.e., the number of confirmed deaths divided by the number of confirmed cases) was observed (45), due to a more effective COVID-19 case tracking system (which identified asymptomatic cases more often than in the first wave) and the refinement of the quality of care provided (46). In Italy, a large vaccination campaign was launched in January 2021 (47), and subsequently the fourth wave showed fewer cases, deaths, and hospitalizations (10, 35). Starting in autumn-winter 2022, the fifth wave reached the highest prevalence values and the lowest lethality rate, due to the emergence of new, less aggressive viral variants, in addition to all the factors mentioned above (10, 36). Finally, on 31st March 2022, the Italian government declared the end of the emergency status (48), and from then on, the subsequent waves became less definable and perceptible, even if more frequent (49). To date, the WHO has announced the end of the COVID-19 pandemic in 2023 because of the reduction in viral morbidity and mortality (50). These temporal dynamics demonstrate the strong contribution of governmental measures and multiple interventions, including pharmaceutical and non-pharmaceutical ones, to pandemic control (51, 52). Our results are consistent with this evidence, showing an improvement in clinical-organizational management after the first wave (Figure 2). In addition, several previous studies confirmed that the implementation of concrete actions in hospitals during the first wave was beneficial for increasing surge capacity and reducing staff workload (53–55). In particular, non-pharmaceutical approaches helped to mitigate the outbreaks; however, their impact may be dynamic, due to variations in implementation and degree of compliance (56).

In this sense, the impact of this study lies in the actions implemented by Spoleto Hospital, that may offer a replicable framework for preparedness planning in future health emergencies, such as: hospitals should develop flexible models for material and human resources that can quickly adapt to fluctuations in demand for care, such as telemedicine and staff rotation (Section 3, 5 and 9); hospitals should design contingency protocols for the transformation of spaces and the separation of clinical pathways (Section 4); hospitals should implement education and training programs for health-care workers on the management of emergencies and the use of PPE (Section 7 and 8). It is important to highlight how the individual and combined effects of these five specific interventions have led to improvements in health system performance. Interestingly, the literature also reports that telemedicine (e.g., email, voice call, video call, text message) can effectively reduce the physical burden on healthcare facilities (57). Along with designing safety pathways (58, 59), maintaining routine primary care (60) and using PPE (61), telemedicine is one of the most effective interventions for preventing the transmission of nosocomial infections (62).

Finally, we developed a multiple-choice hospital worker experience survey for these items. This type of survey is quick, reliable, and easy to code, and captures the meaning behind the experience with accurate responses and reduced bias (63). We then used the SPRIS system. This is an organizational analysis tool previously validated (24), that converts the qualitative survey results into quantitative data, providing a single performance indicator and allowing for direct and objective comparison that can be extended to other subjects studied in different systems and scenarios. In particular, we carried out the analysis at two levels of depth, the first for items and the second for sections, obtaining two performance indexes for each respondent. It should be noted that the performance indexes obtained for the items were similar to those calculated for the sections, but they could not have been the same. This is because the section aggregation level hides the impact of the items. As an example, when the worst items are aggregated into a single section, their impact is smaller and the performance index for the section is higher than that for the item. However, the performance range was always the same. Therefore, the shallower analysis is faster but less accurate, while the deeper analysis is more accurate but less immediate, and the choice depends on the analysis context.

In conclusion, using the SPRIS system we have identified key issues in the response of Spoleto Hospital during the COVID-19 pandemic. Similar elements that influenced the birth of organizational learning have been highlighted by an analysis involving ten hospitals in central and northern Italy (64), in particular: availability of resources (spaces, materials, personnel), consolidated professional relationships, and standardization of protocols and procedures. Moreover, preparedness planning and training and managerial openness are also conducive to a successful COVID-19 pandemic response in six hospitals across Brazil, Canada, France and Japan (65). By this comparison, the perspective adopted and the scenario tested fit current healthcare contexts.

### 4.1 Limitations and future prospectives

Nevertheless, this study has some limitations. First, we considered the study periods as the first to fifth “waves” to simplify the setting of the observational study, which inevitably cannot cover the complex epidemiologic phenomenon. Second, only the hospital unit referents were invited to respond, which were easily accessible. This can be considered a starting point and an acceptable approach when using a qualitative design (28); however, all health-care workers should participate in the interviews. Future wider surveys are needed to fully describe the hospital experience. Third, we used the Likert scale because it allows for quicker and more detailed interpretation of the level of agreement/disagreement than information from open-ended or binary questions, respectively. In addition, as a worded scale, it can capture the respondent’s opinion better than a numerical scale because it has more emotional connotation and objectivity in the meaning of each category. However, the use of the Likert scale is controversial because of the statistical treatment of its data and the level of precision that can be achieved. Finally, the SPRIS system has several limitations (24) (i.e., standardization, not friendly use), but they can be overcome by the continuous application of the SPRIS system contributing to its validation and improvement process.

## 5 Conclusion

This study contributed to the understanding of how a medium-sized hospital can maintain and even improve the quality of care, by revealing a changing pattern in the management of medical facilities during the first five successive waves of COVID-19 pandemic. In the hospital of Spoleto, the health management protocols and processes were successfully monitored and reviewed through performance-based indicators provided by the SPRIS system. This study could be considered as a starting point for the analysis, monitoring and evaluation of new strategies for the management of health-care units during emergency periods.

## Data Availability

The original contributions presented in the study are included in the article/Supplementary material, further inquiries can be directed to the corresponding author.
