# Supplementary figures and images for: The Italian public health response during the pandemic emergency: from qualitative data to the “performance index” of care provided by Spoleto Hospital
Source: Front Public Health. 2025 Jun 16;13:1337375. doi: 10.3389/fpubh.2025.1337375 (PMC12206721; doi:10.3389/fpubh.2025.1337375)

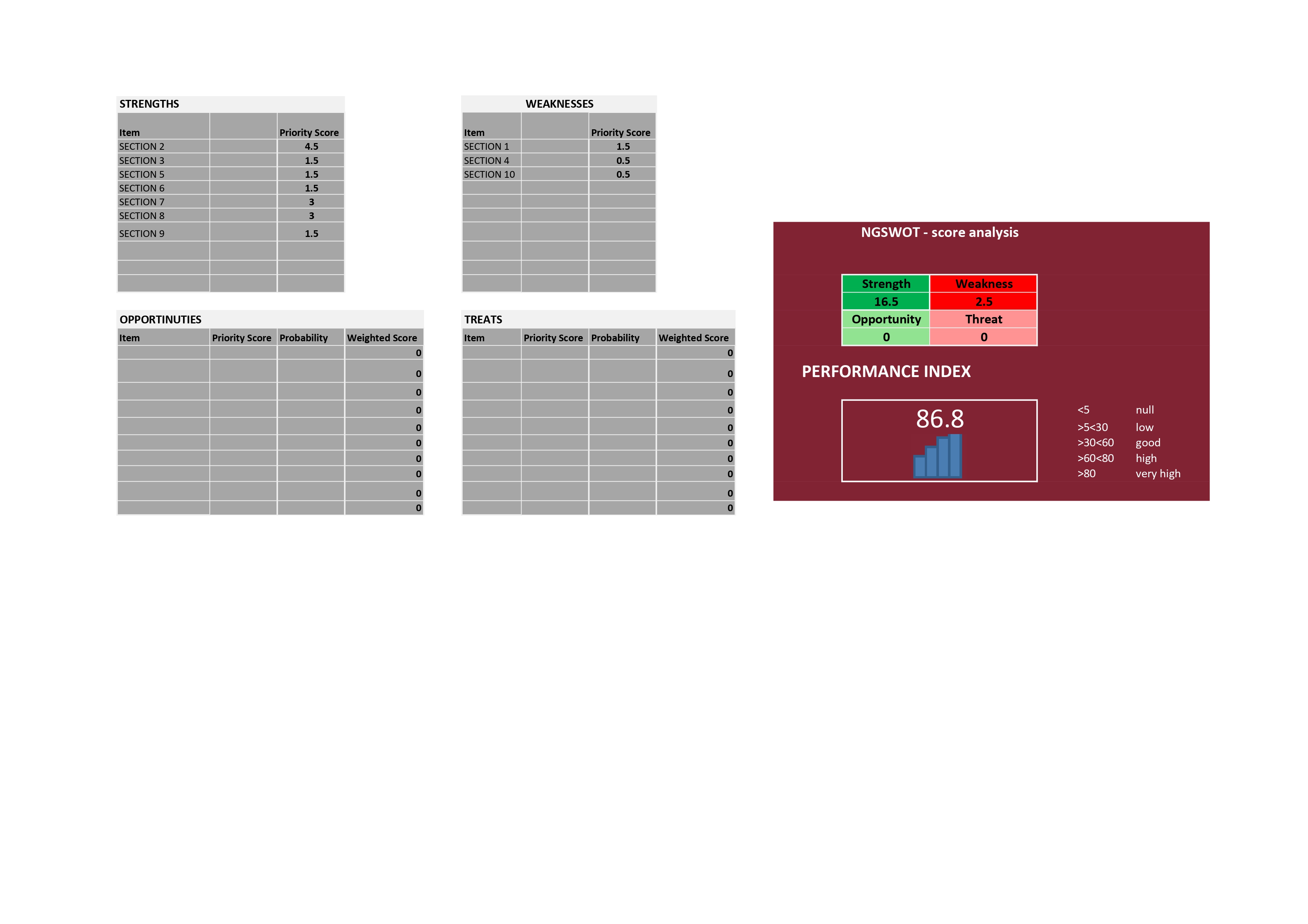

Supplement: Supplementary file 1 [file Image_1.jpg]

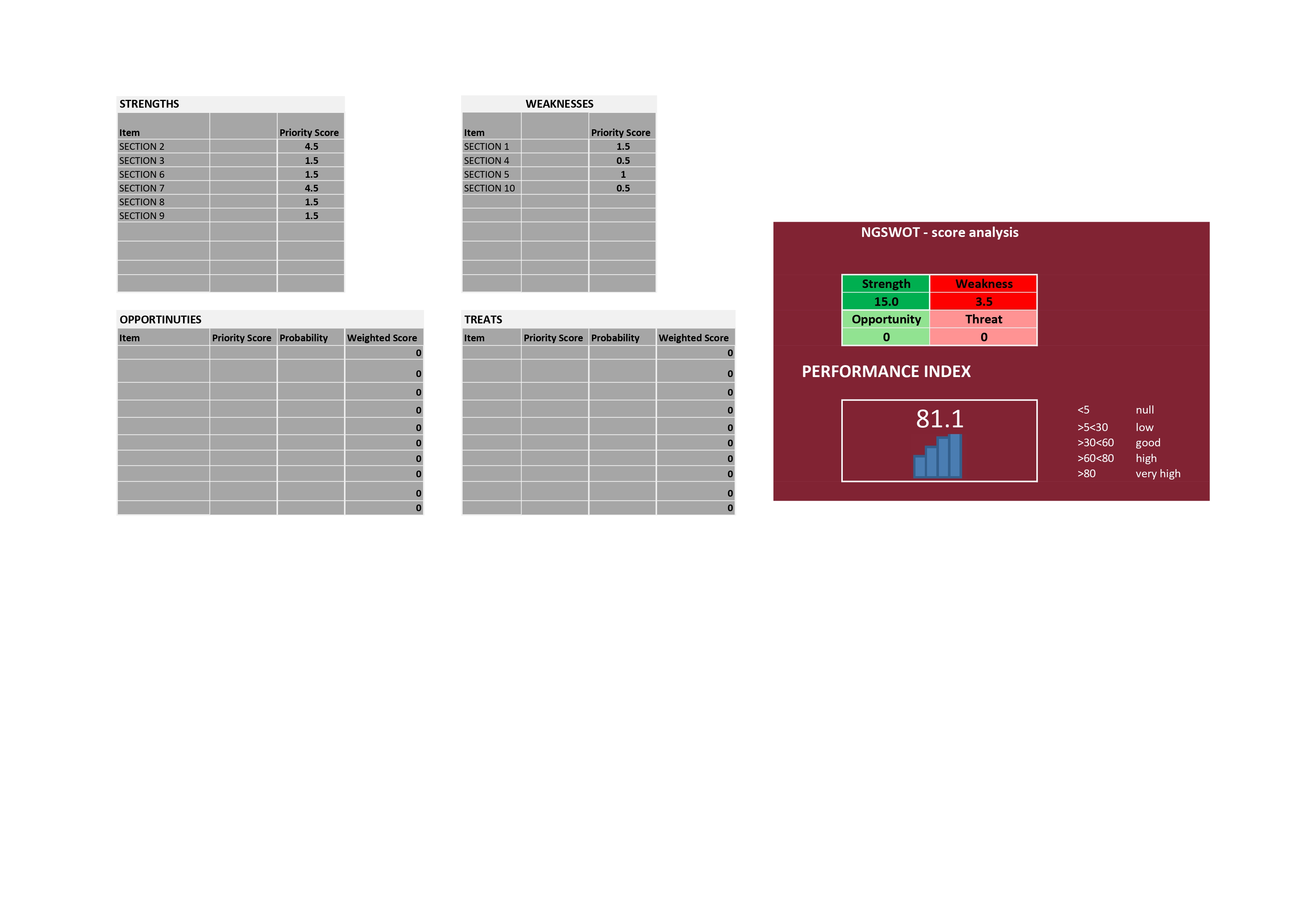

Supplement: Supplementary file 2 [file Image_2.jpg]

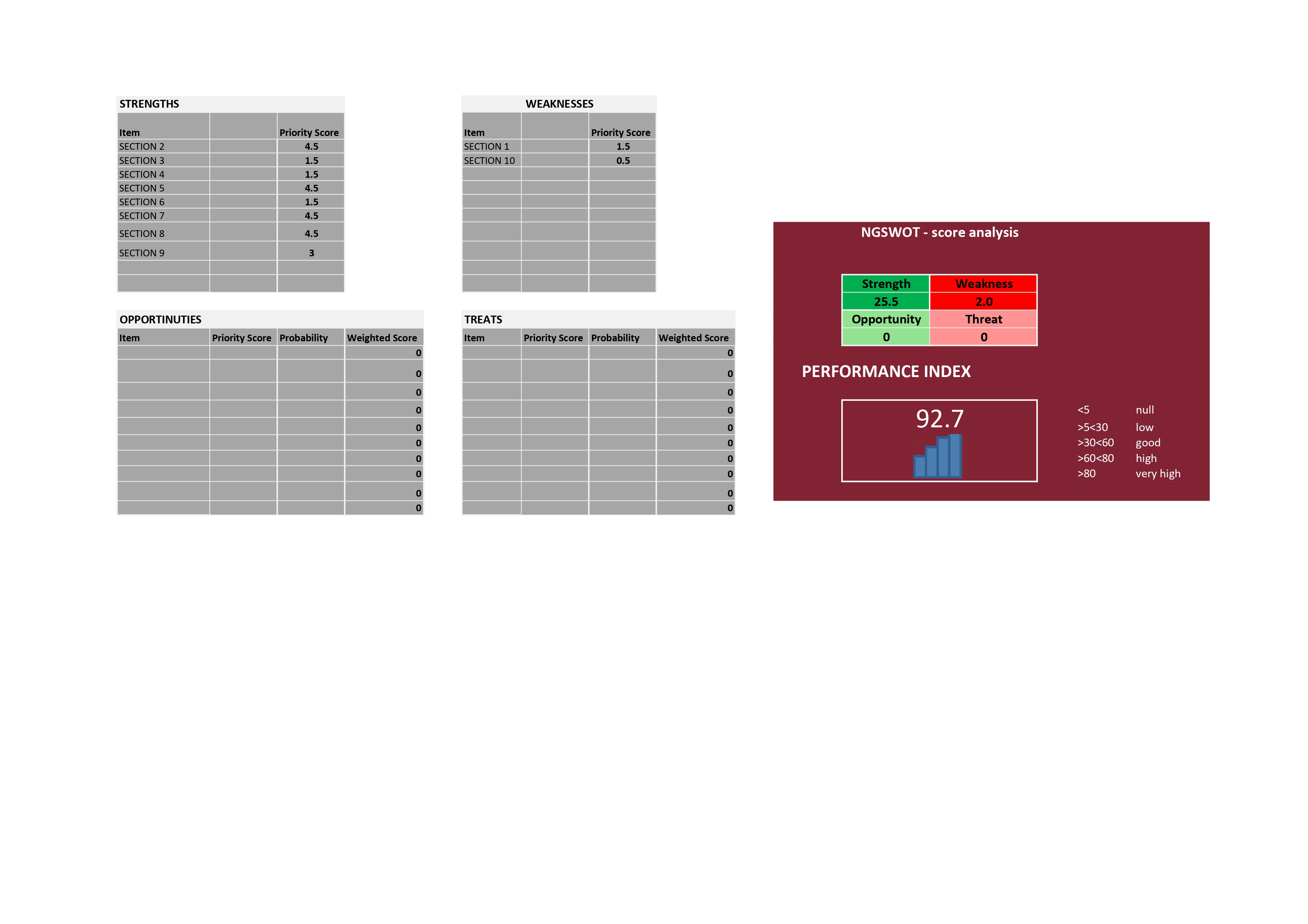

Supplement: Supplementary file 3 [file Image_3.jpg]

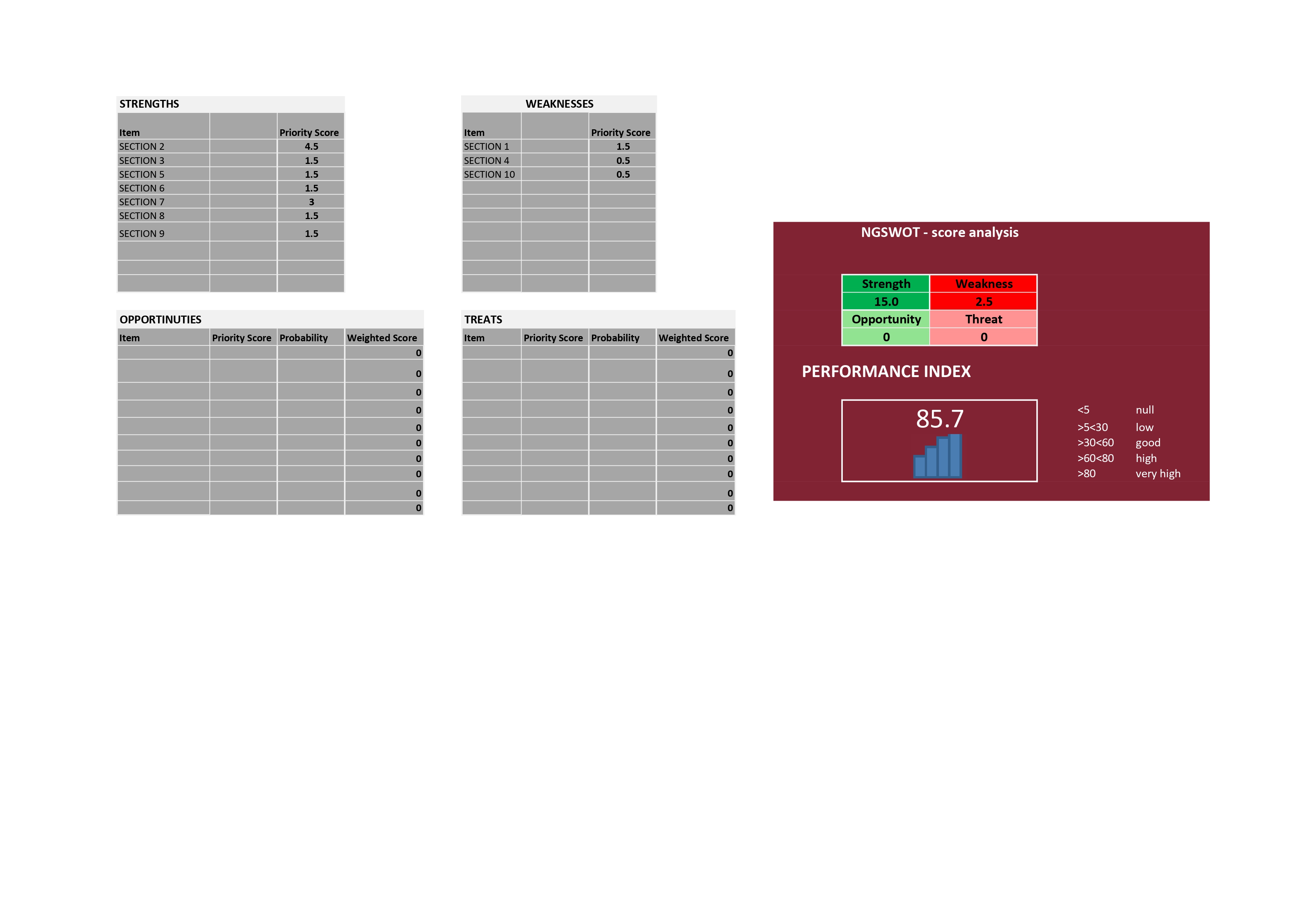

Supplement: Supplementary file 4 [file Image_4.jpg]
