# Supplementary material for: The Italian public health response during the pandemic emergency: from qualitative data to the “performance index” of care provided by Spoleto Hospital
Source: Front Public Health. 2025 Jun 16;13:1337375. doi: 10.3389/fpubh.2025.1337375 (PMC12206721; doi:10.3389/fpubh.2025.1337375)
Supplement: Supplementary file 8 [file Table_4.docx]

**Supplementary Table 4.** **Summary of performances divided into every wave.** Results related to respondents individually.

| **Respondent Aggregation Level** | **Respondent** | **Performance Index (%) per time-point ("wave")** | | | | |  |  |  |
| --- | --- | --- | --- | --- | --- | --- | --- | --- | --- |
|  |  | **i** | **ii** | **iii** | **iv** | **v** |  |  |  |
| for each OU | General Medicine | 13,6 | 93,3 | 83,3 | 85,7 | 75 |  | **Legend of**  **performance ranges** | |
|  | Onco-haematology | 70,6 | 70,6 | 70,6 | 80,2 | 80,2 |  |  |  |
|  | General Surgery | 60 | 63,2 | 55,7 | 51,6 | 63,2 |  | 0 | null |
|  | Obstetrics and Gynecology | 50 | 48,4 | 48,4 | 47,9 | 58,1 |  | 10 | low |
|  | Ophthalmology | 67,1 | 67,1 | 67,1 | 66,3 | 65,4 |  | 20 |  |
|  | Orthopaedic-Traumatology | 49,4 | 50 | 45,6 | 37 | 46,8 |  | 30 |  |
|  | Reanimation | 80,8 | 93,3 | 93,3 | 60 | 72 |  | 40 | good |
|  | Pathological Anatomy | 100 | 100 | 100 | 100 | 100 |  | 50 |  |
|  | Anesthesiology | 61,5 | 96,6 | 83,5 | 80,3 | 86,2 |  | 60 | high |
|  | Angiology | 92,3 | 93,3 | 100 | 100 | 100 |  | 70 |  |
|  | Cardiology | 19,7 | 86,2 | 90 | 93,3 | 88,5 |  | 80 | very high |
|  | Dietetics | 85,7 | 96,6 | 100 | 93,3 | 93,8 |  | 90 |  |
|  | Gastrointestinal Endoscopy | 89,3 | 90 | 90 | 89,3 | 89,3 |  |  |  |
|  | Hepatology | 89,5 | 89,5 | 89,5 | 90,5 | 90,5 |  |  |  |
|  | Analysis Laboratory | 87,8 | 89,4 | 89,4 | 86,8 | 86,8 |  |  |  |
|  | Nephrology and Dialysis | 100 | 100 | 100 | 100 | 100 |  |  |  |
|  | Neurophysiopathology | na | na | na | na | na |  |  |  |
|  | Radiology | 93,8 | 93,8 | 93,8 | 93,8 | 93,8 |  |  |  |
|  | Radiotherapy | 76,6 | 76,6 | 76,6 | 76,6 | 76,6 |  |  |  |
|  | Cardiovascular | 19,7 | 86,2 | 90 | 93,3 | 88,5 |  |  |  |
|  | Rehabilitation | 87,1 | 87,1 | 90 | 93,8 | 93,8 |  |  |  |
|  | Audiology, Phoniatrics and Ear-nose-laryngology | 19,7 | 86,2 | 90 | 93,3 | 88,5 |  |  |  |
|  | General Surgery | 71,4 | 69,5 | 60 | 63,2 | 58,8 |  |  |  |
|  | Orthopaedics | 55,8 | 55,8 | 48,8 | 44,6 | 44,6 |  |  |  |
|  | Paediatrics | 90 | 93,1 | 96,6 | 96,6 | 100 |  |  |  |
|  | Hospital Polyclinics | 52,3 | 66,3 | 66,3 | 48,2 | 48,2 |  |  |  |
|  | Accident and Emergency | 93,8 | 93,8 | 96,8 | 96,8 | 100 |  |  |  |
|  | Pain Therapy | 36,5 | 34,3 | 58,1 | 58,1 | 58,1 |  |  |  |
